# *Enterococcus* isolated from poultry intestine for potential probiotic use

**DOI:** 10.14202/vetworld.2023.1605-1614

**Published:** 2023-08-17

**Authors:** E. Carvajal, S. Contreras, W. Díaz, D. Martinez-Bello, M. McCown, Y. Ardila, María Cristina Vásquez

**Affiliations:** 1 Universidad de Santander, Facultad de Ciencias Médicas y de la Salud, Instituto de Investigaciones Masira, Bucaramanga, Colombia; 2 Universidad de Santander, Facultad de Ciencias Exactas, Naturales y Agropecuarias, Bucaramanga, Colombia; 3 US Army Veterinary Public Health Officer and Zoonotic and Infectious Diseases, University of Florida College of Veterinary Medicine, University of Florida, Gainesville, FL, USA

**Keywords:** *Enterococcus avium*, *Enterococcus faecium*, lactic acid bacteria, probiotics

## Abstract

**Background and Aim:**

To develop species-specific probiotics for poultry, it is ideal to obtain these probiotic microorganisms directly from the intestinal tract of broiler and egg-laying chicks in production environments to ensure adaptation to actual conditions. This study aimed to isolate lactic acid bacteria (LAB) from the intestinal tract of broiler and egg-laying chicks to determine their probiotic potential.

**Materials and Methods:**

Twenty-five Ross-308 broilers and 25 Isa Brown egg-laying chicks were raised until days 42 and 120, respectively; they were housed in an individual poultry building. Lactic acid bacteria were isolated and identified from the small intestine mucus of broiler and layer chicks and then evaluated based on resistance to acidic pH levels, bile salt concentration, and antagonistic activity against wild strains of *Escherichia coli* and *Salmonella* spp. selected strains with probiotic potential were identified by polymerase chain reaction and confirmed by rDNA sequencing.

**Results:**

One hundred and fifty Gram-positive isolates were obtained; 28% (42) were catalase and oxidase negative and biochemical identification was made by crystal system: 76.2% (32) *Enterococcus* spp., 16.6% (7) *Lactococcus* spp., and 7.2% (3) *Streptococcus* spp.; and evaluated for hemolysin production; tolerance to low pH and bile salts, and antagonistic potential were carried out. Molecular characterization yielded 56% (24) *Enterococcus faecium*, and 44% (18) *Enterococcus faecalis*. About 81% (34) of strains were without vancomycin resistance genes criterion.

**Conclusion:**

This study isolated and characterized 36 strains of LAB with probiotic qualities, from the intestines of broiler and egg-laying chicks, selecting *E. faecium*, *Enterococcus avium*, and *Enterococcus casseliflavus*, *Lactococcus garviae* as promising strains for further *in vitro* and *in vivo* research.

## Introduction

Probiotics are toxin-free producer microorganisms, which, if supplied appropriately, balance and maintain gastrointestinal health. In humans, there is evidence of the probiotics’ ability to restore intestinal ecology, reducing the burden of pathogenic microorganisms [1]. Probiotic microorganisms include lactic acid bacteria (LAB), widely known as lactic acid producers from carbohydrate metabolism LAB pathways. These Gram-positive microorganisms are facultative anaerobic fermenters, catalase and oxidase-negative [2]. *Lactobacillus*, *Bifidobacterium*, *Enterococcus*, and *Bacillus* are some of the most frequently used bacterial species as probiotic microorganisms. However, fungi species such as *Aspergillus oryzae* and yeast such as *Candida pintolopesii*, *Saccharomyces boulardii*, and *Saccharomyces cerevisiae* are also employed as probiotics [3].

Some selection criteria for bacteria probiotics are: (a) To be present in the intestinal flora of the animal species of interest, since in this way, they will adapt more efficiently to the gastrointestinal environment in each specie conditions; (b) tolerate low pH conditions, since they must pass through the stomach; (c) tolerate the bile salts action as in the first portion of the small intestine (duodenum) [4]; and (d), recently the International Scientific Association of Probiotics and Prebiotic also established that there must not exist vancomycin resistance genes (VRGs) [5]. Compliance with these criteria and the functionality and safety of probiotic microorganisms used in the industry are regulated and guaranteed by the Food and Agriculture Organization and the World Health Organization [6]. Lactic acid bacteria antagonize pathogenic bacteria by competing on adhesion sites and nutrients as well as producing bacteriocins (proteins and peptides with bactericidal properties) and volatile fatty acids [7]. Benefits in gastrointestinal functioning and strengthening of native intestinal flora in animals supplemented with LAB have been demonstrated in humans [8], fishes [9], pigs [10], goats [11], cattle [12], and birds [13]. Furthermore, some effects on the immune system have been described, such as the modulation of gut-associated lymphoid tissue response by stimulating immunoglobulin A and interferon-gamma production [14, 15], and the activation of CD4 and CD8 T lymphocytes [16]. Poultry production operates under stressful conditions and can promote infectious disease spread due to the high population density, thus affecting health and productivity [17]. To reduce or treat these situations, producers use prophylactic antibiotics; however, the short production periods in poultry are sometimes not enough for antibiotics to be entirely metabolized and then some traces can be found in meat and eggs [18, 19], facilitating the occurrence of bacterial resistance to antibiotics. Therefore, it is necessary to develop more efficient, effective, and innocuous therapeutic and/or nutritional alternatives to reduce the use of antibiotics in animals for human consumption. In some instances, probiotic microorganisms have been derived from native chickens under field conditions, providing the opportunity to incorporate biodiversity into the microorganism collections used for commercial purposes [20], including *Lactobacillus* spp. genus [21, 22]. Following these examples, we raised broiler and egg-laying chicks in field conditions, taking care of not using growth promoters (currently used in commercial poultry food in Colombia), so we can recover and select intestinal microorganisms with probiotic potential.

This study aimed to isolate LAB from the intestinal tract of broiler and egg-laying chicks raised under field conditions and fed without growth promoter to determine their probiotic potential (tolerance to low pH and bile salts, and antagonistic performance against strains of *Escherichia coli* and *Salmonella* spp.), as well as to assess the presence of antibiotic resistance genes.

## Materials and Methods

### Ethical approval

All procedures were designed and carried out in accordance with the guidelines established by the Colombian Animal Protection Regulation on Animal Research (Law 84 of december 27, 1989, National Animal Protection Statute).

### Study period and location

This study was conducted from June 2015 to March 2017. For this study, we used poultry from a rural location in the city of Bucaramanga, province of Santander (Colombia).

### Poultry handling

Twenty-five Ross-308 broiler chicks and 25 Isa Brown egg-laying chicks were bred until days 42 and 120, respectively; they were housed in poultry cages under field conditions in a rural location of the city of Bucaramanga, province of Santander (Colombia). The birds were fed and watered *ad libitum*, and lighting and heating were provided according to breed requirements (Ross-308 and Isa Brown Manual). All animals were fed with antibiotic-free food whose formulation was based on the nutritional requirements established by the Spanish Federation for the Development of Animal Nutrition.

### Isolation and identification of LAB

Birds were sacrificed by cervical deviation at weeks 1, 2, 3, 4, and 5 for broilers and 2, 6, 17, and 35 for egg-laying chicks, and the intestines were removed from the body of each bird according to the necropsy method described by Martínez-Acevedo [23]. Deep mucous membrane scraping was performed in the duodenum, jejunum, and ileum; subsequently serial dilutions were made up to 10–10 and last five dilutions of each intestinal segment were seeded in Man Rogosa Sharpe (MRS) agar (Merck, Darmstadt, Germany), to obtain axenic cultures. The bacteria obtained were selected according to their morphological characteristics. Gram staining, catalase test, and oxidase test were performed and LAB were identified using the semiautomated system Crystal for Gram-positive (BBL™ Crystal™ Gram Positive ID Kit (BD, New Jersey, USA).

### Isolates hemolytic activity

Twenty-five milliliters of blood agar was served in Petri dishes, three wells were made using a sterile element, and each well was inoculated with 50 μL of axenic culture of the strains previously isolated by Miller *et al*. [24] in Brain Heart Infusion broth and incubated for 48 h at 37°C with AnaeroGen® (Oxoid, UK), 13% CO_2_. Afterward, the halos produced were read according to the type of hemolysis (Alpha, Beta, and Gamma) [25].

### Resistance of isolates to low pH and bile tolerance

Isolates were inoculated in test tubes with MRS broth, whose pH was modified with 1N HCl at the following values: pH2, pH3, pH4, and pH5. They were incubated at 41°C for 4 h until a turbidity of 0.5 was obtained in Farlanthe scale [26]; three inocula of 50 μL were made on MRS agar and incubated for 72 h with AnaeroGen system 13% CO_2_ [27]. The percentage of resistance was calculated using the Kociubinski formula [28], by comparing colony-forming units per microliter of the control (no treat culture) (CCFU/μL), and the treated culture (pHCFU/μL) tested.


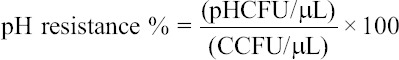


### Resistance of isolates to bile salt

Isolates were inoculated in test tubes with MRS broth modified with chicken bile at: 0.1%, 0.15%, 0.2%, and 0.3% concentrations; incubated at 41°C for 4 h, then three inocula of 50 μL were made on MRS agar (Merck) and incubated for 72 h with the AnaeroGensystem (Hampshire, UK), at a concentration of 13% of CO_2_ [27]. The percentage of survival was calculated by modifying the equation of [28].


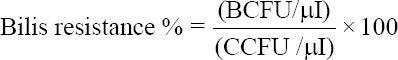


### Antagonistic activity

Four strains of *E. coli* and two of *Salmonella* spp. were seeded on soybean trypticase agar (STA) plates, five wells were made in each plate, then 50 μL of cultures of each isolated strain were inoculated in each well, incubated at 41°C by 72 h with the AnaeroGen system 13% CO_2_ [27]; inhibition halos were measured [29].

### DNA extraction

DNA extraction was carried out from pure cells suspended in sterile water with the MOBIO Ultraclean Microbial DNA Isolation commercial kit (Irvine, CA, USA) was used, following the manufacturer’s instructions. At the end of the procedure, the concentration and purity of DNA obtained by spectrophotometry were verified in Nanodrop equipment and its subsequent visualization was carried out in 1% agarose gel.

### Polymerase chain reaction (PCR) and bioinformatics analysis

Fragments amplification was carried out with 16S universal initiators (1492R-27F). The PCR assays were performed in 25 μL of reaction mixture containing 21 μL of Master mix, 1 μL of reverse primer, 1 μL of reverse primer, and 2 μL of genomic DNA, under the following conditions: A denaturation initial step at 94°C for 3 min, followed by 30 denaturation cycles at 94°C for 45 s, annealing at 63°C for 40 s and extension at 72°C for 90 s, and finally a 72°C extension stage for 10 min. The amplified PCR products (2 μL) were visualized in 1.5% agarose gel, and documented by a light transilluminator. Polymerase chain reaction products were sequenced by Corpogen (Bogotá) in alliance with Macrogen (Korea). The sequences were subjected to a Blast in the National Center for Biotechnology Information database and subsequently analyzed.

### *VanA* and *VanB* genes determination

To determine the presence of *Van A* and *Van B* genes, vancomycin PCR duplex protocol [30]. *VanA* gene Primer Forward EA1 (+): 5’-GGGAAAACGACAATTGC-3’, *VanA* gene Primer Reverse EA2 (-): 5’-GTACAATGCGGCCGTTA-3, product: 732 pb. *VanB* Primer Forward: 5’- ACGGAATGGGAAGCCGA -3’, Primer Reverse: 5’- TGCACCCGATTTCGTTC -3’, and Product: 647 pb.

### Statistical analysis

Continuous variables are expressed as means (standard deviations) and categorical variables as percentages. The percentage resistance to pH levels (2, 3, 4, and 5) and bile concentrations (0.1, 0.15, 0.2, and 0.3) are expressed as mean and standard deviation, and the antagonistic behavior of the isolated strains to challenging strains of *E. coli* and *Salmonella* is expressed as the percentage of antagonistic isolates by strain.

Cluster analysis using Gower distance for dissimilarity matrix was accomplished for the isolated strains for the resistance percentage to pH and bile salts concentration, and for the antagonistic behavior to challenging strains of *E. coli* and *Salmonella*. We employed the divisive clustering method, starting from all isolated strains and deriving clusters based on similar pH and bile salt resistance characteristics for one side and antagonistic behavior to challenging strains for the other side. Dendrograms were elaborated based on clusters obtained using the divisive clustering method. Then, we describe the average resistance percentage by cluster to the pH and bile salt levels, presenting the isolated strains in every cluster. In addition, we show the isolated strains’ average percentage of antagonistic behavior to challenge strains by cluster and the isolated strains’ names by cluster. The R software version 4.1 was used for the computations [31].

## Results

### Isolation and screening

One hundred and fifty isolates were initially obtained. Gram staining, catalase and oxidase tests were performed. Forty-two isolates that were negative for catalase and oxidase tests and classified as Gram-positive were used in the experiments below. Identification was carried out with crystal system for Gram-positive (BBL Crystal™ Gram-positive ID Kit- (Oxoid) and three genus were initially identified (*Enterococcus* spp., *Lactococcus* spp., and *Streptococcus* spp.) and distributed as follows; 4 (9.5%) *Enterococcus avium*, 4 (9.5%) *Enterococcus casseliflavus*, 1 (2.4%) *Enterococcus durans*, 5 (12%) *Enterococcus faecalis*, 18 (42.8%) *Enterococcus faecium*, 1 (2.4%) *Lactococcus garviae*, 2 (4.8%) *Lactococcus lactis*, 3 (7.1%) *Lactococcus rafinolactis*, and 3 (7.1%) *Streptococcus vestibularis*.

### Hemolytic activity

A hemolysin production test was performed on 42 strains, of which 6 (14.3%) strains presented alpha and/or beta hemolysis. Only bacteria of the genus *Enterococcus* spp. showed some type of hemolysis; all hemolytic isolates were dismissed.

### Low pH and bile salt resistance

Table-1 shows the mean and standard deviation of the dilution and the resistance percentage to the pH levels and to the bile salt concentration levels. Most of the strains were isolated from broiler chicks (32) versus laying chicks (4). From broiler chicks, we isolated eight strains from duodenum, 12 strains from jejunum, and 12 strains from Ileum, while from laying chicks, we isolated two strains from duodenum, and one strain in jejunum and ileum. The resistance percentage to pH 2 ranged from 20% for *L. rafinolactis* in broiler chick’s ileum, to 70% for *S. vestibularis* in duodenum. The resistance percentage to pH 3 ranged from 39.7% for *E. faecalis* strains in broiler chick’s ileum to 85% for *L. lactis lactis* in broiler chicks’ jejunum. The resistance percentage to pH 4 and pH 5 was above 50% in all isolated strains for both pH levels. The average resistance percentage to four bile concentrations is shown in Table-1. The resistance percentage to a bilis concentration of 0.3 ranged from 25% for *L. rafinolactis* in duodenum and *L. lactis cremoris* and *S. vestibularis* in jejunum to 90% for *E. faecalis* in duodeum, while for a 0.2 bile concentration, it ranged from 35% for *L. rafinolactis* in duodenum to 70% *L. rafinolactis* in ileum.

**Table-1:** Number of selected bacterial strains for minor intestine portion, dilution [mean +– (SD)], the resistance percentage [mean +– (SD)] to four pH levels (2, 3, 4, and 5) and resistance percentage [mean +– (SD)] to four bile concentration 0.1%, 0.15%, 0.2% and 0.3%.

Strain	n	Dilution	pH 2	pH 3	pH 4	pH 5	Bile 0.1	Bile 0.15	Bile 0.2	Bile 0.3
Broiler chick										
Duodenum										
*Enterococcus faecalis*	1	8	30	45	65	90	85	60	52	90
*Enterococcus faecium*	3	8.3 (1.5)	28.3 (2.9)	51.7 (2.9)	80 (10)	58.3 (10.4)	78.3 (7.6)	68.3 (7.6)	60 (0.1)	63.3 (5.8)
*Lactococcus garviae*	1	6	50	60	75	65	70	75	60	30
*Lactococcus rafinolactis*	2	8.5 (2.1)	45 (7.1)	70 (14.1)	77.5 (3.5)	80 (21.2)	50 (35.4)	45 (35.4)	35 (21.2)	25 (21.2)
*Streptococcus vestibularis*	1	9	70	65	80	50	70	50	50	45
Jejunum										
*Enterococcus avium*	1	6	39	50	90	88	85	80	62	88
*Enterococcus casseliflavus*	2	9 (1.4)	32.5 (9.2)	42.5 (10.6)	81 (12.7)	89 (1.4)	92.5 (3.5)	72.5 (10.6)	52.5 (3.5)	89 (1.4)
*Enterococcus faecium*	6	7.2 (1.6)	30.8 (10.2)	50.5 (7.4)	68.3 (10.3)	70.8 (18.6)	81.7 (5.2)	66.7 (8.2)	60.3 (8.5)	60 (16.7)
*Lactococcus lactis cremoris*	1	7	45	60	80	60	85	60	52	25
*Lactococcus lactis lactis*	1	7	50	85	90	90	80	65	52	45
*Streptococcus vestibularis*	1	9	45	60	80	60	85	60	52	25
Ileum										
*Enterococcus avium*	3	8.7 (1.2)	38 (7.5)	56.7 (5.8)	84.7 (4.6)	87.7 (2.5)	89 (3.6)	81.7 (2.9)	60.7 (8.1)	87.7 (2.5)
*Enterococcus faecalis*	3	7.3 (0.6)	21.7 (2.9)	39.7 (0.6)	59 (3.6)	89 (3.6)	83.3 (5.8)	80 (10)	67.7 (17.2)	88.3 (2.9)
*Enterococcus faecium*	5	8 (1.9)	40 (20)	55 (9.4)	71.4 (6.7)	61 (17.5)	77 (4.51)	62 (8.4)	53.6 (5.9)	59 (19.5)
*Lactococcus rafinolactis*	1	9	20	40	80	90	90	70	70	60
Laying chick										
Duodenum										
*Enterococcus faecalis*	1	9	25	40	60	80	85	85	70	80
*Streptococcus vestibularis*	1	9	50	60	75	65	70	75	60	30
Jejunum										
*Lactococcus lactis lactis*	1	6	25	48	50	80	70	75	60	70
Ileum										
*Enterococcus durans*	1	9	30	50	80	70	80	80	70	50

**Figure-1:**
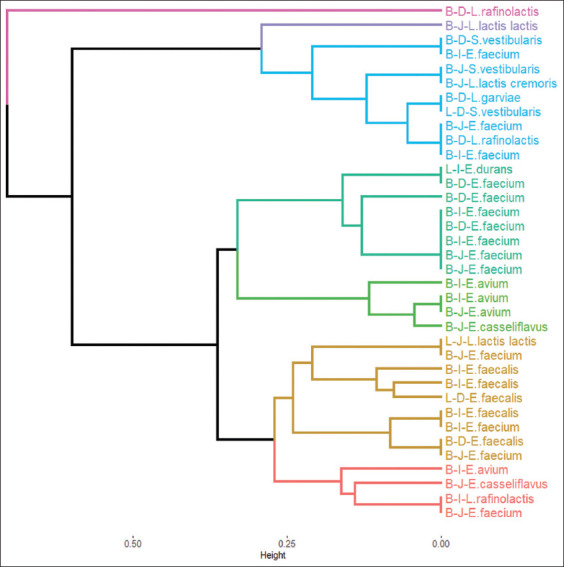
Dendrogram of the isolated strains clusters selected by pH levels and bile concentrations. B=Broiler chick, L=Egg laying chick, D=Duodenum, J=Jejunum, I=Ileum.

The dendrogram obtained from the clustering method for the combined clustering effects of pH levels and bile salt levels is shown in Figure-1. Based on the within clusters sums of squares, we select seven clusters shown in Figure-1.

Table-2 displays the average percentage resistance to four pH levels and four bile salt concentrations by cluster. We observed that cluster 3 shows the highest average resistance to pH 2 (41%) and the highest average resistance to bile salts concentration of 0.3 (87%).Table-3 presents the isolated strains by cluster and from that we found that cluster 3 contains *E. casseliflavus* and *E. avium* from Broiler chick’s Jejunum, and two strains of *E. avium* from broiler chick’s Ileum.

**Table-2:** Average of the percentage resistance to four pH levels [mean +– (SD)] and four bile concentration [mean +– (SD)] by cluster.

Cluster	n	pH 2	pH 3	pH 4	pH 5	B 0.1	B0.15	B0.2	B0.3
1	4	24 (4.9)	43.8 (11.1)	78.5 (4.4)	90 (0)	90.5 (1)	71.3 (6.3)	65.8 (7.2)	75 (17.3)
2	9	53.3 (9.7)	61.1 (2.2)	77.2 (2.6)	60.6 (6.3)	75 (6.1)	64.4 (9.8)	52.7 (4.2)	35.6 (8.1)
3	4	40.5 (3)	52.5 (5)	88 (4)	87.3 (1.5)	88.8 (4.8)	81.3 (2.5)	56.5 (6.4)	87.3 (1.5)
4	9	24.4 (3.9)	42.8 (3.7)	58.8 (5.8)	86.3 (5.1)	82.8 (3.6)	75 (10.6)	62.8 (12.6)	79.4 (17)
5	8	29.4 (1.8)	53.1 (2.6)	75 (7.6)	55.6 (9)	78.1 (5.3)	65 (7.1)	60 (0)	62.5 (4.6)
6	1	40	80	80	95	25	20	20	10
7	1	50	85	90	90	80	65	52	45

**Table-3:** Isolated strains selected from the percentage resistance to four pH levels [mean +– (SD)] and four bile concentrations [mean +– (SD)] by cluster.

Cluster ID	Line	Location	Strains
1	Broiler chick	Jejunum	*Enterococcus faecium, Enterococcus casseliflavus*
		Ileum	*Lactococcus rafinolactis, Enterococcus avium*
2	Broiler chick	Duodenum	*Lactococcus rafinolactis, Lactococcus garviae, Streptococcus vestibularis*
		Jejunum	*Lactococcus lactis cremoris, Enterococcus faecium, Streptococcus vestibularis*
		Ileum	*Enterococcus faecium (2 strains)*
	Laying chick	Duodenum	*Streptococcus vestibularis*
3	Broiler chick	Jejunum	*Enterococcus casseliflavus, Enterococcus avium*
		Ileum	*Enterococcus avium (2 strains)*
4	Broiler chick	Duodenum	*Enterococcus faecalis*
		Jejunum	*Enterococcus faecium (2 strains)*
		Ileum	*Enterococcus faecium, Enterococcus faecalis (3 strains)*
	Laying chick	Duodenum	*Enterococcus faecalis*
		Jejunum	*Lactococcus lactis lactis*
5	Broiler chick	Duodenum	*Enterococcus faecium (3 strains)*
		Jejunum	*Enterococcus faecium (2 strains)*
		Ileum	*Enterococcus faecium (2 strains)*
	Laying chick	Ileum	*Enterococcus durans*
6	Broiler chick	Duodenum	*Lactococcus rafinolactis*
7	Broiler chick	Jejunum	*Lactococcus lactis lactis*

### Antagonistic activity

**Figure-2:**
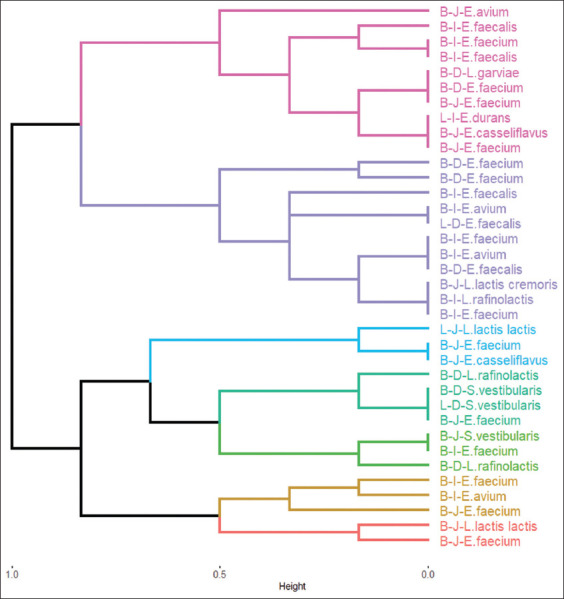
Dendrogram of the isolated strains clusters selected by antagonistic behavior to challenging *Escherichia coli* and *Salmonella* strains. B=Broiler chick, L=Egg laying chick, D=Duodenum, J=Jejunum, I=Ileum.

The antagonistic activity was performed against (4) *E. coli* and (2) *Salmonella* spp. wild-type strains. Table-4 shows the antagonistic characteristics of isolated strains to challenging strains of *E*. *coli* and *Salmonella* spp. *E. durans* in laying chicks’ ileum and *E. avium* in broiler chicks’ jejunum showed antagonistic behavior to five challenging strains, *L. garviae* in broiler chicks’ duodenum, and *E. avium* in broiler chicks’ jejunum to four challenging strains. In contrast, *E. faecium* strains in broiler chicks’ jejunum showed antagonistic behavior to all challenging strains.Figure-2 shows the dendrogram for the clustering method, for the antagonistic characteristics of the isolated strains to challenging strains of *E. coli* and *Salmonella* spp. We again established seven clusters based on the within cluster sum of squares. Table-5 shows the average percentage of isolated strains antagonist to the challenging strains.

**Table-4:** Percentage of isolated strains showing antagonistic behavior to the challenging strains of *Escherichia coli* and *Salmonella* by strain, region of the minor intestine, and chick type (broiler or laying).

Strain	n	*E. coli* 32	*E. coli* 40	*E. coli* 42	*E. coli* 46	*Salmonella* 1	*Salmonella* 2
Broiler							
Duodenum							
*Enterococcus faecalis*	1	-	100	-	100	-	-
*Enterococcus faecium*	3	100	33.3	-	100	33.3	33.3
*Lactococcus garviae*	1	100	-	-	100	100	100
*Lactococcus rafinolactis*	2	50	100	-	-	50	-
*Streptococcus vestibularis*	1	-	100	100	-	100	-
Jejunum							
*Enterococcus avium*	1	100	-	100	100	100	100
*Enterococcus casseliflavus*	2	50	100	50	50	50	50
*Enterococcus faecium*	6	83.3	66.7	66.7	50	83.3	33.3
*Lactococcus lactis cremoris*	1	-	-	-	100	-	-
*Lactococcus lactis lactis*	1	-	-	100	100	100	-
*Streptococcus vestibularis*	1	100	100	-	-	100	-
Ileum							
*Enterococcus avium*	3	-	100	33.3	100	-	33.3
*Enterococcus faecalis*	3	-	66.7	-	100	100	66.7
*Enterococcus faecium*	5	40	80	20	80	40	20
*Lactococcus rafinolactis*	1	-	-	-	100	-	-
Laying chick							
Duodenum							
*Enterococcus faecalis*	1	-	100	-	100	-	100
*Streptococcus vestibularis*	1	-	100	100	-	100	-
Jejunum							
*Lactococcus lactis lactis*	1	-	-	100	-	-	-
Ileum							
*Enterococcus durans*	1	100	100	-	100	100	100

**Table-5:** Mean percentage of antagonistic behavior by challenging strains of *Escherichia coli* and *Salmonella* by cluster.

Cluster ID	n	*E. coli* 32	*E. coli* 40	*E. coli* 42	*E. coli* 46	*Salmonella* 1	*Salmonella* 2
1	2	-	50	100	100	100	-
2	11	18	64	-	100	9	18
3	3	100	100	-	-	67	-
4	3	-	67	100	-	-	-
5	4	-	100	75	-	100	-
6	10	70	50	10	100	100	100
7	3	67	100	100	100	33	-

From Table-5, we observed that cluster 6 contains 10 isolated strains having antagonistic behavior in 70% to *E. coli* 32, 50% to *E. coli* 40, 100% to *E. coli* 46, 100% to *Salmonella* 1, and 100% to *Salmonella* 2, making those strains in this cluster to be strong candidates to be considered as probiotic strains, where isolated strains in cluster 6 are comprised by *E. faecium*, *L. garviae* in Broiler chicks Duodenum, *E. faecium* (two strains), *E. casseliflavus*, *E. avium* in broiler chicks jejunum, *E. faecalis* (two strains), *E. faecium* in broiler chicks ileum, and *E. durans* in laying chicks ileum (Table-6). Finally, we would like to add that from the most resistant cluster 3 to pH and bile salts levels, we found *E. casseliflavus* in broiler chicks’ duodenum and *E. avium* in broiler chicks jejunum, which are also included in the clustering results for cluster 6 in the antagonistic behavior to the challenger strains of *E. coli* and *Salmonella* spp.

**Table-6:** Selected strains for the antagonistic behavior to challenging strains of *Escherichia coli* and *Salmonella* by cluster.

Cluster Id	Line	Location	Strains
1	Broiler chick	Jejunum	*Enterococcus faecium, Lactococcus lactis lactis*
2	Broiler chick	Duodenum	*Enterococcus faecium (2 strains), Enterococcus faecalis*
		Jejunum	*Lactococcus lactis cremoris*
		Ileum	*Enterococcus faecium (2 strains), Enterococcus faecalis, Lactococcus rafinolactis, Enterococcus avium (2 strains)*
	Laying chick	Duodenum	*Enterococcus faecalis*
3	Broiler chick	Duodenum	*Lactococcus rafinolactis*
		Jejunum	*Streptococcus vestibularis*
		Ileum	*Enterococcus faecium*
4	Broiler chick	Jejunum	*Enterococcus casseliflavus, Enterococcus faecium*
	Laying chick	Jejunum	*Lactococcus lactis lactis*
5	Broiler chick	Duodenum	*Lactococcus rafinolactis, Streptococcus vestibularis*
	Laying chick	Duodenum	*Streptococcus vestibularis*
	Broiler chick	Jejunum	*Enterococcus faecium*
6	Broiler chick	Duodenum	*Enterococcus faecium, Lactococcus garviae*
		Jejunum	*Enterococcus faecium (2 strains), Enterococcus casseliflavus, Enterococcus avium*
		Ileum	*Enterococcus faecalis (2 strains), Enterococcus faecium*
	Laying chick	Ileum	*Enterococcus durans*
7	Broiler chick	Ileum	*Enterococcus avium, Enterococcus faecium*
		Jejunum	*Enterococcus faecium*

### Vancomycin resistance

**Figure-3:**
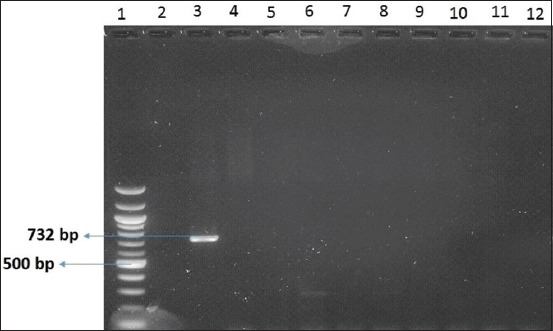
Example of the polymerase chain reaction test results to demonstrate vancomycin resistance genes in nine strains isolated from broiler and egg-laying chicks.

Duplex PCR was performed to identify VRGs. No VRG was found in the 25 strains (Figure-3).

### Molecular characterization

Twenty-five enterococci strains without VRG were characterized by sequencing, and bioinformatics analysis showed that all 25 strains were enterococci bacteria: 14 (56%) *E. faecium* and 11 (44%) *E. faecalis*. All useful isolated enterococci strains came from broilers, except by one (4%) from egg-laying chicks (Table-2).

## Discussion

This study shows the selection process of field strains candidates to be probiotic bacterial strains isolated from broiler and laying chicks in Colombia. Based on the resistance to pH and bile salts concentration, we selected *E. casseliflavus* and *E. avium* from broiler chicks jejunum, and two strains of *E. avium* from broiler chicks Ileum, while based on the antagonistic activity to *E. coli* and *Salmonella* strains, we selected *E. faecium*, *L. garviae* in Broiler chicks duodenum, *E. faecium* (two strains), *E. casseliflavus*, *E. avium* in broiler chicks jejunum, *E. faecalis* (two strains), *E. faecium* in broiler chicks ileum, and *E. durans* in laying chicks ileum. Combining both characteristics, we found that *E. avium* and *E. casseliflavus* from duodenum obtained from broiler chicks are strong candidates to explore further *in vivo* activity as probiotic agents in Broiler chicks. *Enterococcus faecium* has shown probiotic activity in isolations from silage [32], and it has been evaluated in piglets for managing weaning diarrhea [33], and the immunomodulating activity in the nematode model [34]. Chicken meat-associated isolates of *Enterococcus* are often multidrug-resistant, closely related to pathogenic strains, and harbor perturbing virulence factors, representing enterococci evolving into drug-resistant human pathogens [35]. We found that the strains in this study were negative for harboring VRGs. Although the probiotic potential has been explored in Enterococci, we also found studies showing no effect on the productive responses but influencing positively over the feed and chick cost [36]. *Enterococcus faecium* are probiotic candidates which can be applied in commercial scenarios to improve poultry performance and control of pathogens, hence decreasing further transmission to humans [37]. *Enterococcus faecium*, *E. faecalis*, and *E. durans* have been employed as probiotics to prevent subclinical necrotic enteritis (SNE) [38, 39]. Comprehensive evaluation showed that *E. faecium* could be a candidate as a probiotic strain used in humans or animals, with the potential to restrain the effect of *Salmonella* strains [40–42]. We also found evidence in the literature that *E. faecium* strains isolated from chicken are alternatives to antibiotics to reduce feed conversion rate in broiler chicken, possibly mediated by increasing the villus height of the intestinal epithelium, so the mechanisms involve keeping healthy the intestinal architecture of the epithelium [43]. In this study, we provide the microorganisms population biodiversity from the small intestine parts (duodenum, jejunum, and ileum) from broiler and egg-laying chicks raised in field conditions without the use of growth promoters from a rural location in a Colombian town, isolating multi-genus populations such as *E. faecalis*, *E. faecium* [44], *E. avium*, *E. casseliflavus*, and *L. garviae*, similar to the findings of Neveling *et al*. [44]. Finally, we conclude that we have found a set of potential LAB strains to evaluate *in vivo* the probiotic effect on the commercial and productive features in broiler and egg-laying chicks. The study’s findings demonstrate the value of the research in this field and that future research must be accomplished to develop and advance alternatives to prophylactic probiotics in poultry production.

## Conclusion

In this study, we isolated and characterized probiotic potential LAB strains from poultry. Isolates were identified as *E. faecium*, *E. avium*, *E. casseliflavus*, and *L. garviae*, some promising microorganism strains for further research and use as probiotics in animal production. However, the actual relevance of our findings lies in the possibility of finding microorganisms with the potential to reduce the administration of antibiotics in animal industries, such as the poultry industry, where antibiotics are widely accepted and improve animal and human health, according to the One Health concept.

## Authors’ Contributions

YA, EC, SC, and MCV: Study design, fieldwork, data collection, and sample processing. YA, DM, WD, and MM: Analysis and interpretation of the data. MCV, YA, DM, WD, MM, and EC: Drafted and revised the manuscript. All authors have read, reviewed, and approved the final manuscript.
